# Overexpression of GSK-3β in Adult Tet-OFF GSK-3β Transgenic Mice, and Not During Embryonic or Postnatal Development, Induces Tau Phosphorylation, Neurodegeneration and Learning Deficits

**DOI:** 10.3389/fnmol.2020.561470

**Published:** 2020-09-10

**Authors:** Alberto Rodríguez-Matellán, Jesús Avila, Félix Hernández

**Affiliations:** ^1^Centro de Biología Molecular Severo Ochoa (CSIC-UAM), Madrid, Spain; ^2^Network Center for Biomedical Research in Neurodegenerative Diseases (CIBERNED), Madrid, Spain

**Keywords:** Alzheimer’s disease, GSK-3β, neurodegeneration, tau, transgenic mice

## Abstract

GSK-3β or tau-kinase I is particularly abundant in the central nervous system (CNS), playing a key role in the pathogenesis of Alzheimer’s disease (AD). Accordingly, transgenic mouse models overexpressing this kinase recapitulate some aspects of this disease, such as tau hyperphosphorylation, neuronal death, and microgliosis. These alterations have been studied in mouse models showing GSK-3β overexpression from birth. In this case, some of these alterations may be due to adaptations that occur during development. Here we explored the potential of the Tet-OFF conditional system in the murine CamKIIα-tTA/GSK-3β model to increase the activity of GSK-3β only during adulthood. To this end, the overexpression of GSK-3β remained OFF during embryonic and postnatal development by administration of doxycycline in drinking water for 6 months, while it was turned ON in adult animals by removal of the treatment for 6 months. In these conditions, the CamKIIα-tTA/GSK-3β mouse is characterized by an increase in phosphorylated tau, cell death, and microgliosis. Furthermore, the increase in GSK-3β expression in the adult animals triggered a cognitive deficit, as determined through the hippocampus-dependent object recognition test (OR). These results demonstrate that the GSK-3β plays a key role in AD and that previously published data with other transgenic models are neither caused by or a consequence of adaptations to high levels of the enzyme during development.

## Introduction

Alzheimer’s disease (AD) was one of the first diseases reported involving an alteration in the activity of the GSK-3β enzyme (Hernandez et al., 2013). In this regard, it was suggested that GSK-3β mediates tau hyperphosphorylation, β-amyloid-induced neurotoxicity, and the mutagenic pathogenic effects of presenilin 1 (Jope et al., 2007). Increased expression of GSK-3β was also observed in the post-mortem analysis of brains of patients with AD compared to samples from age-matched controls (Pei et al., 1999; Leroy et al., 2007). GSK-3β has a central role in development, controlling key neuron processes such as neuronal proliferation and differentiation, neurite outgrowth, polarity, and cytoskeleton stability. GSK-3β also regulates synaptic plasticity, and neurotransmitter release and receptor trafficking (Beurel et al., 2015; Jaworski et al., 2019).

The first murine models of GSK-3β focused on the effects of the overexpression of this enzyme on the central nervous system (CNS) during the development of AD. To this end, promoters with neuronal expression were used. A characteristic common to all these promoters is that they are active from the last days of intrauterine development. Therefore, some of the parameters studied and published may not be due to the increase in GSK-3β caused by AD but to adaptations that occur during development under elevated expression of the kinase. Interestingly, an increase in GSK3β levels is observed during postnatal days 5–15 and then declines during the rest of life (Takahashi et al., 2000; Yu et al., 2009).

In 1997, Brownlees and collaborators generated the first two murine GSK-3β transgenic models: one of wild-type GSK-3β and the other expressing a mutant form of GSK-3β in which serine-9 was mutated to alanine (S9A), which results in a more active form since phosphorylation inactivation of serine 9 is prevented (Jope and Johnson, 2004). These transgenes were under the transcriptional control of the ubiquitous promoter of the murine sarcoma virus (MSV) or the specific neuronal promoter of the murine neurofilament light chain (NF-L; Brownlees et al., 1997). Despite detecting the two mRNA transgenes, no substantial increase in total GSK-3β activity was observed with either of the two forms of GSK-3β. Those authors postulated that this observation is attributable to the toxicity caused by GSK-3β overexpression during embryonic and postnatal development of the CNS.

The second transgenic animal to be published also expressed the S9A mutant form of the kinase, this time under the control of the modified thy1 murine promoter, which allows transgene expression only postnatally and only in neurons (Spittaels et al., 2000). This mouse shows an increase in GSK-3β activity, as well as an increase in phosphorylation of the tau protein, but only during adulthood (7–8 months). The second round of characterization of this mouse revealed a significant decrease in the weight and overall volume of the brain, with the greatest reduction occurring in the cerebral cortex and not being attributable to apoptotic phenomena (Spittaels et al., 2002). Despite the increase in tau phosphorylation and decrease in brain weight, GSK-3β[S9A] mice performed normally to find the hidden platform in the Morris water maze but their swim speed was significantly lower than that of non-transgenic mice (Spittaels et al., 2002). Li et al. (2004) used mice expressing the same mutant GSK-3β[S9A] isoform, but under the control of the promoter of the human platelet-derived growth factor (PDGF) B-chain that began at day 15 of gestation, and reached the greatest expression around the time of birth (Sasahara et al., 1991). This promoter drove expression of the kinase mainly to the cortex and hippocampus, with an increase in tau phosphorylation, as detected by the AT8 antibody. No evidence of apoptosis was detected in these animals.

Given the postulated lethality of the embryonic overexpression of GSK-3β (Brownlees et al., 1997), as well as the known role of this kinase in development—the response of neural progenitors to differentiate depends largely on GSK3 activity (Holowacz et al., 2013)—a transgenic mouse was generated using the conditional system regulated by tetracycline (TetO/GSK-3β; Lucas et al., 2001). This animal, which shows conditional overexpression of GSK-3β in neurons (under the CamKIIα promoter), recapitulates the neuropathological aspects of AD (Lucas et al., 2001). Thus, in previous studies, overexpression of this enzyme from the neonatal period led to an increase in phosphorylated tau, enhanced apoptosis, as well as activation of reactive gliosis. These cellular alterations triggered learning deficits (Hernández et al., 2002; Engel et al., 2006b).

Animals generated through the use of the tetracycline-regulated system allow us to explore reversal of the generated phenotype and are thus valuable models to test the neuroprotective effect of specific GSK-3β inhibitors. Thus, transgenic shutdown in symptomatic mice by doxycycline administration leads to normal GSK-3 activity, normal levels of phospho-tau, decreased neuronal death, and suppression of cognitive deficits, thereby further supporting the potential of inhibitors of GSK-3 in AD (Engel et al., 2006b).

Here we sought to exploit the CamKIIα-tTA/GSK-3β mouse model to increase the activity of GSK-3β only during adulthood, in contrast to our previous work in which activity was increased from birth (Lucas et al., 2001; Hernández et al., 2002; Engel et al., 2006b) or with other transgenic models (Brownlees et al., 1997; Spittaels et al., 2002). These previous studies have shown that an increase in GSK3β neonatally has consequences; however, the effect of an increase in GSK-3β activity only during adulthood has not been addressed. The experimental approach proposed herein allowed us to generate a model that more faithfully replicates the conditions that occur in AD, characterized by manifestation at advanced ages. To this end, we administered doxycycline in drinking water during the first 6 months of the mouse’s life to maintain the system inhibited. We then withdrew this treatment, thus inducing GSK-3β overexpression, for an additional 6 months, until sacrifice.

## Materials and Methods

### Animals

All the animals were housed in the Animal Facility at the Centro de Biología Molecular “Severo Ochoa” (CBMSO) under a 12 h light/12 h dark cycle in a temperature-controlled environment and were provided with food and water *ad libitum*. All the animal care protocols complied with national legislation (RD 53/2013) and the guidelines of the European Commission for the housing and care of laboratory animals (revised in Appendix A of the Council of Europe Convention ETS123). All procedures were approved by the Bio-Ethics Committee of CBMSO, the Spanish Research Council (CSIC), and the Consejería de Medioambiente de la Comunidad de Madrid (PROEX-412/15).

The CamKIIα-tTA/GSK-3β model (referred to in the rest of the manuscript as BG6 model; Lucas et al., 2001) overexpresses GSK-3β in cells expressing calmodulin kinase IIα. This overexpression is repressible by the administration of dissolved doxycycline in drinking water (Tet-OFF system). It is a double transgenic mouse generated from the crossing of the line containing the transgene with the repressible transactivator under the control of calmodulin kinase IIα promoter (line B) with the BitetO-β-Gal/GSK-3β transgenic mouse line (line G6). This tTA line was chosen to allow restricted and conditional expression in the CNS, with particularly high expression in the forebrain (Mayford et al., 1996). Line G6 presents a construct with the GSK-3β coding sequences, with the Myc epitope at the N-terminal end and the β-galactosidase (β-gal) marker protein. For the transcription of both genes to occur, the transactivator with the operating sequence must bind.

We repressed GSK-3β overexpression in transgenic BG6 mice by administering doxycycline in drinking water for 6 months (2 mg/ml), in an attempt to more faithfully reproduce what happens in AD (characteristic disease of advanced ages). Subsequently, the antibiotic was removed from the drinking water and the animals were sacrificed at 12 months of age (animals BG6OFF/ON). Also, we examined BG6 mice administered doxycycline in drinking water for 12 months (animals BG6OFF). Animals in which the system was active since birth (BG6ON animals) were also analyzed. As controls of these models, animals of the BitetO β-Gal GSK-3β line (G6 line) were used under the three conditions described above. A time scheme of the experiments illustrating when was the application of doxycycline started and when completed is shown in Supplementary Figure S1.

### Object Recognition Test

We used 12-month-old mice (both sexes) and in the case of mice with doxycycline treatment, this was given during the behavioral experiments. The object recognition test (OR) was performed as previously described (Engel et al., 2006b), with some modifications.

#### Day 1

The first day of the test is devoted to familiarization. The mice were habituated for 10 min in an empty open-topped methacrylate box measuring 45 × 45 cm with opaque vertical walls.

#### Day 2

On the second day of the test, the animals were introduced in the same box for 5 min with two identical objects (A and B). Both objects were placed on the floor equidistant along the axis of the box and 12.5 cm from the nearest wall. At the end of the 5 min, the mice were removed from the box, which was then cleaned with 70% ethanol to eliminate the smell. Two hours later, each mouse was placed back in the box with one of the old objects (object A), and a new one (object C). The position of object C was the same as that occupied by object B in the previous test, and the time given for the recognition test was also 5 min. A distance of less than 2 cm from the mouse’s head to the object was considered as recognition. The time (tA and tC) that the animal spent exploring the two objects (objects A and C, respectively) was recorded. The memory index (MI), defined as the ratio of the time spent exploring the novel object over the time spent exploring both objects, MI = [tC/(tA + tC)] × 100, was used to measure nonspatial memory.

### Sacrifice and Tissue Processing

Mice were anesthetized using an intraperitoneal injection of pentobarbital (Dolethal, 60 mg/kg bW) and perfused transcardially with saline. Brains were extracted and separated into two hemispheres. One hemisphere was removed and fixed in 4% paraformaldehyde in a 0.1 M phosphate solution (PB; pH = 7.4) overnight at 4°C. The next day, the hemispheres were washed three times with the 0.1 M phosphate solution, included in a mixture of 10% sucrose and 4% agarose, and then cut in the sagittal plane using a vibratome (Leica VT2100S). The sections obtained (50-μm thick) were stored at −20°C and cryoprotected in glycol (solution composed of 30% ethylene glycol, 30% glycerol, and 10% phosphate buffer).

The other hemisphere was rapidly dissected, obtaining the hippocampus and other structures of interest, which were immediately frozen in liquid nitrogen and stored at −70°C.

### Immunohistochemistry

The sections were washed with PBS to remove the cryoprotective solution. Subsequently, they were immersed in H_2_O_2_ to 0.33% in PBS for 30 min to block the activity of endogenous peroxidase. Sections were placed in a blocking solution (PBS with 0.5% bovine fetal serum, 0.3% Triton X-100 and 1% BSA) for 1 h and incubated overnight at 4°C with the corresponding primary antibody diluted in the blocking solution: mouse anti-β-galactosidase (1/5,000, Promega); mouse anti-PHF1 (1/200, anti-tau protein phosphorylated in ser396/404, 1/100, Peter Davis (Greenberg and Davies, 1990); mouse anti-caspase-3 (1/100, Invitrogen); mouse anti-Iba-1 (1/500, Wako); goat anti-Doublecortin (DCX; 1/500, Santa Cruz); or anti-calretinin (1/200, Swant).

The next day, the sections were washed three times for 10 min with PBS. They were then incubated first with the biotinylated secondary antibody and then with the avidin-catalase complex using the Elite Vectastain kit (SIGMAFASTTM DAB, Sigma, D4293). The developing reaction was performed using diaminobenzidine for approximately 10 min. Finally, the sections were placed in slides using FluorSave (Calbiochem, Merck Millipore) as a mounting medium. Images were taken using an Olympus BX41 transmitted light microscope that uses an Olympus ColorView IIIu CCD docked camera.

### Immunofluorescence

After performing three washes with PB 0.1N, the sections were incubated with the following primary antibodies in the blocking solution (1% BSA and 1% Triton X-100 in 0.1 N PB) at 4°C for 72 h: mouse anti-β-galactosidase (1/5,000, Promega) and rabbit anti-NeuN (1/1,000, Millipore). Next, five washes were performed with this same blocking solution, and the sections were incubated with the corresponding secondary antibodies conjugated with Alexa fluorophores at 4°C with gentle agitation for 24 h (1/1,000, Molecular Probes). Finally, three washes were performed with 0.1 N PB with DAPI diluted 1:5,000 for 10 min, and another three additional washes were performed with PB 0.1 N. The sections were placed on slides using FluorSave. Images were taken using an LSM710 laser and multi-scan scanning microscope coupled to an inverted AxioObserver microscope (Zeiss).

### Quantifications

Antibodies against doublecortin, calretinin, Iba-1, caspase-3, and PHF-1 were quantified by counting the number of positive cells located in four sections of the dentate gyrus (with an average area of 0.15 mm^2^). For atrophy measurement, areas of the dentate gyrus were delineated and measured using the Methamorph image-analysis system. The slices used for analysis were 30 μm hippocampal sagittal sections (matching Figure 114 of Paxinos and Franklin, 2001) at 1.56 mm concerning the midline. For the quantification of activated Iba-1+ cells, a criterion based on cell morphology was used. Activated but non-phagocytic (type C) and a phagocytic state (type D) cells were counted as activated microglia (see Cuadrado et al., 2018 for a full morphology description).

### Western Blot

Brain tissue preserved at −70°C was homogenized to obtain the extracts using a glass-glass potter. The homogenization buffer used was 50 mM Tris-HCl pH 7.4, 1% NP-40, 150 mM NaCl, 1 mM EDTA, 0.25% sodium deoxycholate, and phosphatase inhibitors (1 mM NaF, Na_3_VO_4_ 1 mM and 1 μM okadaic acid) plus the COMPLETE TM protease inhibitor cocktail (Roche). Next, the protein concentration of each homogenate was determined by the Bradford method (Bradford, 1976), using BSA to perform the standard curve. Finally, the SDS-PAGE buffer (250 mM Tris pH 6.8; 4% SDS, 10% glycerol; 2% β-mercaptoethanol and 0.0006% bromophenol blue) was added to the protein extracts obtained. The extracts were boiled in a thermoblock at 100°C for 5 min. Fifty microgram of protein per well was loaded from each sample. The proteins were separated on 10% acrylamide/bisacrylamide gels in the presence of SDS at 120 mV for approximately 1 h. Those present in the gel were transferred to nitrocellulose membranes (Schleicher and Schuell, Keene, NH, USA) at an amperage of 0.15 A for 45 min, using the Bio-Rad Mini-Protean system. Subsequently, the membranes were blocked using 5% milk powder (w/v) in 0.1% Tween PBS (v/v) for 40 min. The membranes were then washed twice with 0.1% PBS Tween-20 (v/v) under stirring for 10 min. Finally, they were incubated with the appropriate primary antibody for 1 h: mouse anti-β-galactosidase (1/5,000, Promega) or mouse anti-GAPDH (1/5,000, Abcam). Protein expression was detected using the secondary goat anti-mouse antibody (1/10,000; Dako) conjugated to HRP. After performing two 10-min washes in the wash solution, the immunoreactive proteins were detected using the ECL (Enhanced Chemiluminescence Detection System, Amersham).

### Statistical Analysis

Statistical analysis was performed with GraphPad Prism software, Version 5.01. The data are presented as mean values ± S.E. Statistical analyses of data were performed by applying a Student’s *t*-test for each statistical comparison. *p* < 0.05 values were considered significant.

## Results

### BG6 Mice Do Not Show Changes in Body or Brain Weight

**Figure 1:**
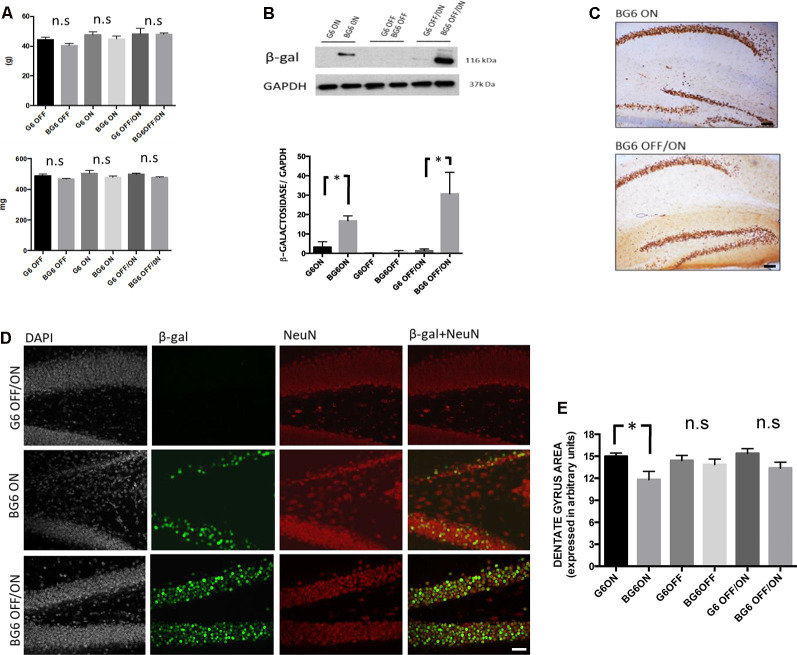
The pattern of transgene expression in mice with the different genotypes and conditions used.** (A)** Bodyweight (G6OFF, BG6OFF, G6ON, BG6ON, G6OFF/ON, and BG6 OFF/ON) measured in grams (g). Brain weights are measured in milligrams (mg). The data shown are the mean values with the SEM; n.s = not significant, *n* = 4–7 animals per group. **(B)** Pattern of hippocampal β-galactosidase (β-gal) expression with GAPDH as a loading control for the hippocampus of the animals tested. The histogram shows β-gal/GAPDH levels in the different genotypes and conditions used. Note the significant increase in the hippocampus in BG6ON mice (*p* = 0.016) and BG6OFF/ON mice (*p* = 0, 026) respect to their G6 controls (*n* = 3 animals per group). **(C)** Expression of β-gal in the hippocampus. Images obtained for immunohistochemistry against β-gal in the hippocampus of BG6ON and BG6OFF/ON animals. **(D)** In the upper row, a dentate gyrus representative of the G6OFF/ON genotype, with a density of nuclei (DAPI) and mature granular neurons (NeuN) typical of a healthy animal and absence of β-gal labeling. In the middle row, a dentate gyrus representative of the BG6ON genotype (with overexpression of GSK-3β for 12 months). We observed degeneration of the dentate gyrus both through nucleus labeling (DAPI) and the limited number of granular neurons (NeuN). Moderate expression of the β-gal marker occurred, being limited to granular neurons (β-gal + NeuN). In the lower row, a dentate gyrus representative of the BG6OFF/ON genotype (overexpression of GSK-3β induced in adult ages). Abundant nuclear density (DAPI) and mature granular neurons (NeuN) are observed, as well as a greater expression of the β-gal in the granular layer (β-gal + NeuN). **(E)** Quantitation (*n* = 6) of the atrophy of dentate gyrus determined as described in the “Materials and Methods” section for the mice of each condition and genotype (G6OFF, BG6OFF, G6ON, BG6ON, G6OFF/ON, and BG6OFF/ON). For quantifications, the data represented are the mean values with the SEM and analyzed with a two-tailed unpaired Student’s *t*-test. *p < 0.05; n.s = not significant. Scale bars = 100 μm.

We first measured the body and brain weight of the animal models used, since overexpression of hypothalamic GSK-3β affects food intake (Benzler et al., 2012). Also, the administration of doxycycline for long periods to keep the Tet-Off system repressed can influence animal weight, as reported for several antibiotics (Furlong et al., 2019). However, the induction of GSK-3β overexpression in adulthood did not lead to significant differences in body weight (Figure 1A). Next, we weighed the brain, the organ overexpressing the kinase. Again, no significant differences were observed in animals with induced GSK-3β overexpression in adulthood or in those that had had the system turned ON from birth (Figure 1A).

### Western Blot and Immunohistochemical Analysis of GSK-3β Expression in the BG6 Model

To determine GSK-3β overexpression, we used a construct with β-galactosidase (β-gal) as a reporter protein. Western blot analysis revealed transgene activation in the hippocampal samples of BG6OFF/ON animals (*p* = 0.026, Figure 1B), thereby showing that the overexpression of this protein is inducible in adult ages. Evidence of overexpression was also found in animals with the system turned ON from birth (mice BG6ON, *p* = 0.016). Also, G6 mice showed a modest expression of the β-Gal reporter in the hippocampus as demonstrated by western blotting (Figure 1B).

Immunohistochemical studies showed considerable β-gal expression in the dentate gyrus of BG6OFF/ON mice. These animals registered greater expression that the BG6ON animals (Figure 1C). We also observed a strong pattern of β-gal expression in the striatum in both conditions (Supplementary Figure S2). However, the distribution of the protein in the cortex differed. In this regard, BG6ON animals showed an overexpression pattern in all cortical layers, except layer IV. However, in BG6OFF/ON animals, the distribution of β-gal was limited to layers II/III, the number of β-gal+ nuclei in the rest of the layers being scarce (Supplementary Figure S2). Given that tau protein pathology similar to that found in AD occurs in the hippocampus (Lucas et al., 2001), we turned our attention to confirming GSK-3β overexpression in granular neurons in the dentate gyrus. Analysis of the colocalization of β-gal with NeuN revealed that the system was ON in granular neurons in BG6OFF/ON animals (Figure 1D). Additionally, in the immunohistochemical analysis of the preparations (Figures 1C,D), we also observed a substantial decrease in the thickness of the dentate gyrus in BG6ON mice as was previously described for BG6ON mice (Lucas et al., 2001; Engel et al., 2006c). Quantitation of the atrophy of dentate gyrus for mice of each condition and genotype demonstrated that atrophy is present in BG6ON mice (*p* = 0.041, Figure 1E), although this is not observed in BG6OFF/ON mice (Figure 1E), suggesting that adult dentate gyrus is less susceptible to neurodegeneration or that at least 12 months (as in BG6ON mice) are necessary to observe atrophy of the dentate gyrus.

### Increased Hyperphosphorylation of Tau Protein in Granular Neurons of the Dentate Gyrus

**Figure 2:**
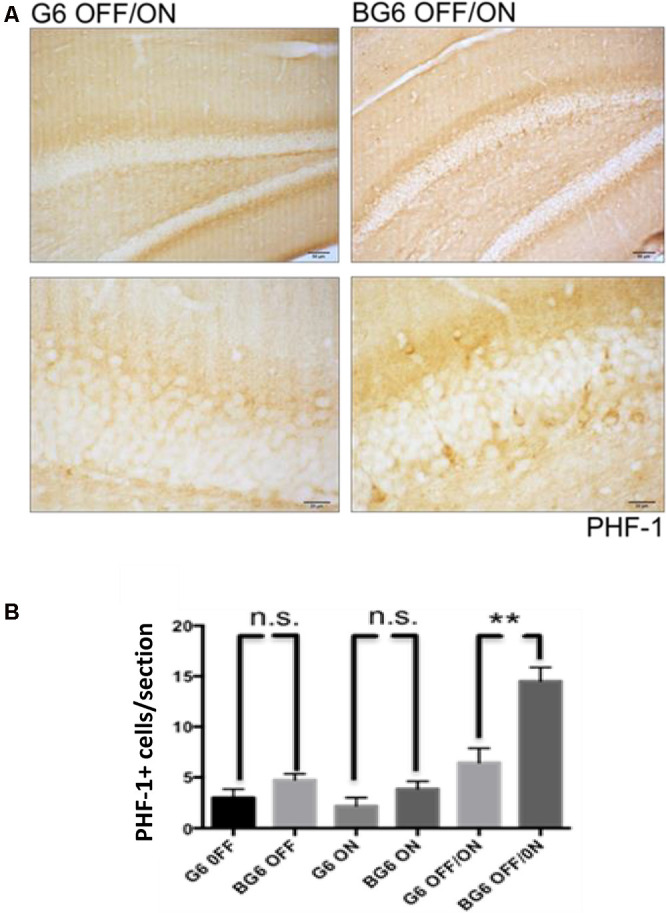
Effect of GSK-3β overexpression on tau phosphorylation in the dentate gyrus. **(A)** Representative images with the corresponding amplification for the PHF-1 antibody in the dentate gyrus of G6OFF/ON and BG6OFF/ON animals. **(B)** Quantification of the number of PHF-1+ cells located in the dentate gyrus of each section for the mice of each condition and genotype (G6OFF, BG6OFF, G6ON, BG6ON, G6OFF/ON, and BG6OFF/ON). For quantifications, the data represented are the mean values with the SEM, and analyzed with two-tailed unpaired Student’s *t*-test; n.s. = not significant, **p < 0.01. Scale bars = 50 μm and 20 μm, *n* = 4–7 animals per group.

Immunohistochemistry against tau epitopes phosphorylated by GSK-3β revealed highly significant differences between the dentate gyrus of G6OFF/ON animals and BG6OFF/ON counterparts (*p* = 0.005, Figure 2). Interestingly, there was no statistical difference between PHF-1 expression in BG6ON mice with the system ON for 12 months and their G6ON controls. An increase in the number of PHF-1+ cells in the dentate gyrus of BG6ON mice has been described in young animals (less than 3 months of age; Lucas et al., 2001). Here we show that this increase does not occur in 12 months old BG6ON animals. This observation could be explained by the death of those neurons with phosphorylated tau and the atrophy described in Figure 1E.

### GSK-3β Overexpression Is Accompanied by an Increase in Caspase-3+ Cells and Microgliosis

**Figure 3:**
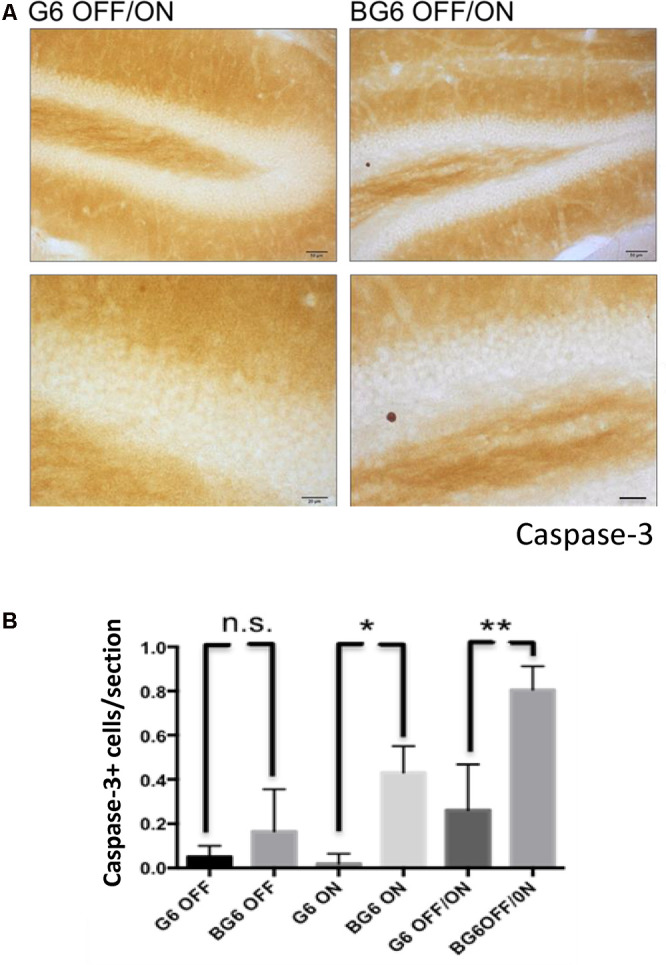
Effect of GSK-3β overexpression on cell death in the dentate gyrus in adult mice. **(A)** Representative images using the proteolyzed caspase-3 antibody with its corresponding amplification for G6OFF/ON and BG6OFF/ON mice. **(B)** Quantifications of caspase-3+ cells of animals of the different genotypes and conditions (G6OFF, BG6OFF, G6ON, BG6ON, G6OFF/ON, and BG6 OFF/ON) obtained by determining the number of positive cells in the dentate gyrus of each section For quantifications, the data represented are the mean values with the SEM, and analyzed with two-tailed unpaired Student’s *t*-test; n.s. = not significant, *p < 0.05, **p < 0.01, Scale bars = 50 and 20 μm, *n* = 4–7 animals per group.

Increased GSK-3 activity triggers programmed cell death (Bijur and Jope, 2001; Gomez-Sintes et al., 2011). Previous data obtained by our group from mice overexpressing GSK3-β in neurons from birth demonstrated elevated apoptosis in the dentate gyrus (Lucas et al., 2001). In the present study, we observed an increase in BG6ON and BG6OFF/ON mice compared with their respective G6 controls (*p* = 0.010 and 0.0086, respectively, Figure 3) indicating that an increase in GSK-3β expression induces cell death by apoptosis.

**Figure 4:**
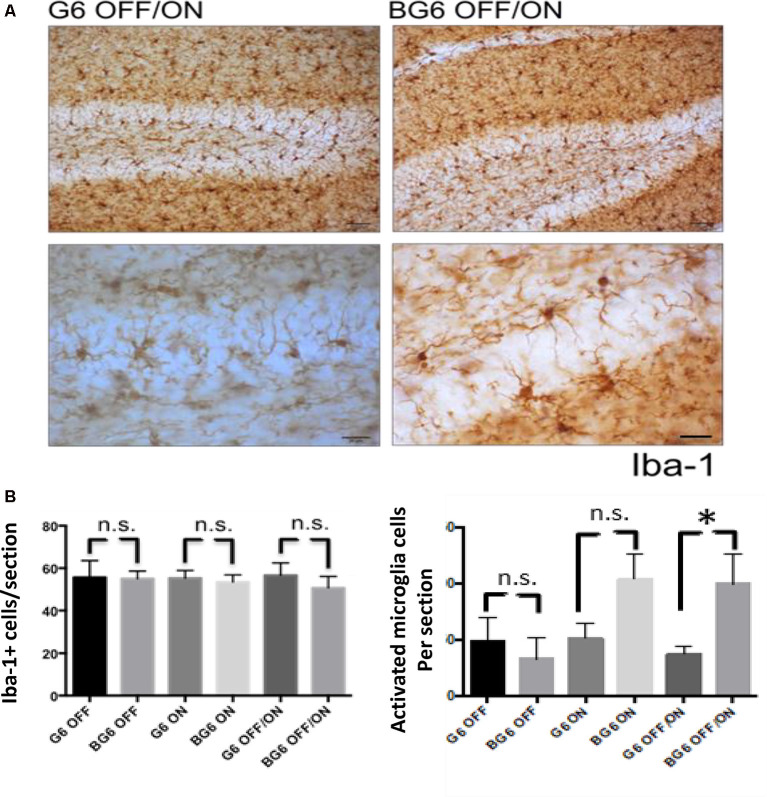
Effect of neuronal GSK-3β overexpression on microgliosis in adult mice. **(A)** Representative images using the iba-1 antibody for G6OFF/ON and BG6OFF/ON mice. **(B)** Quantifications of total Iba-1+ cells of animals of the different genotypes and conditions (G6OFF, BG6OFF, G6ON, BG6ON, G6OFF/ON, and BG6 OFF/ON) obtained by determining the number of positive cells in the dentate gyrus of each section. Bottom right, quantifications of Iba-1+ cells with the morphology of activated microglia of animals of the different genotypes and conditions, obtained by determining the number of positive cells in the dentate gyrus of each section. For quantifications, the data represented are the mean values with the SEM, and analyzed with two-tailed unpaired Student’s *t*-test; *p < 0.05, n.s. = not significant. Scale bars = 50 and 20 μm, *n* = 4–7 animals per group.

To study the levels of microglia, we used the iba-1 antibody (Figure 4). We found no differences in the total number of iba-1+ cells in the dentate gyrus of the BG6OFF/ON animals compared with any of the other conditions (Figure 4B). However, regarding the levels of activated microglia, significant differences were detected between the animals in which the system was induced in adulthood and their internal controls (*p* = 0.037, Figure 4B). BG6ON mice tended to show increased levels of activated microglia, although not in a statistically significant manner (*p* = 0.095, Figure 4B).

### GSK-3β Overexpression Is Accompanied by an Increase in Adult Hippocampal Neurogenesis

**Figure 5:**
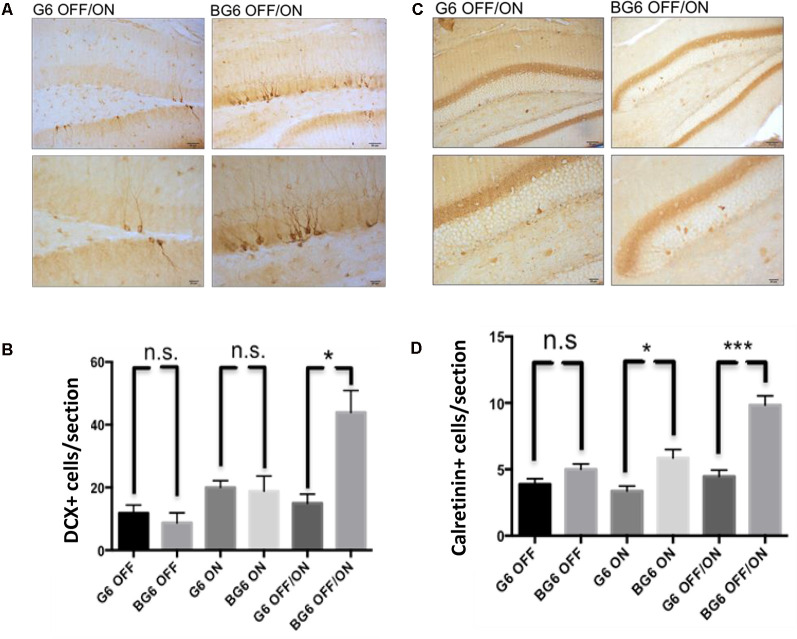
Effect of GSK-3β overexpression on adult hippocampal neurogenesis. **(A)** Representative images of the neurogenic doublecortin marker in the dentate gyrus of G6OFF/ON and BG6OFF/ON animals with their corresponding amplification. **(B)** Quantification of the total number of DCX+ cells by determining the number of positive cells in the granular layer of the dentate gyrus in each section in the animals of the different genotypes and conditions (G6OFF, BG6OFF, G6ON, BG6ON, G6OFF/ON, and BG6 OFF/ON). **(C)** Representative images of the immature calretinin neuron marker in the dentate gyrus of G6OFF/ON and BG6OFF/ON animals with their corresponding amplification. **(D)** Quantification of the total number of calretinin+ cells by determining the number of positive cells in the granular layer of the dentate gyrus in each section in animals of the different genotypes and conditions. For quantifications, the data represented are the mean values with the SEM, and analyzed with two-tailed unpaired Student’s *t*-test; n.s. = not significant; *p < 0.05; ***p < 0.001. Scale bars = 50 and 20 μm, *n* = 4–7 animals per group.

Given that most of the alterations observed in our animal model were analyzed in the hippocampal dentate gyrus and that this region is characterized by the presence of the phenomenon known as adult hippocampal neurogenesis, we sought to study this process. We previously reported an increase in doublecortin+ cells in animals aged 3–6 months with the system turned ON from birth concerning controls (Fuster-Matanzo et al., 2013). In the present study, we found a high increase in doublecortin+ cells in BG6OFF/ON animals concerning their internal controls, thereby confirming that overexpression of GSK-3β during adulthood affects neurogenesis in the dentate gyrus (*p* = 0.013, Figures 5A,B). No differences in this process were found between BG6ON animals (Figure 5B). At this point, we would like to highlight that previous studies had shown an increase in the number of doublecortin+ cells but are relatively young animals. In 15-month-old animals, this increase was no longer observed (Fuster-Matanzo, 2011).

We extended our study on neurogenesis in this model by analyzing calretinin (Figures 5C,D), a calcium transport protein present in immature neurons. Highly significant differences were detected between animals in which GSK-3β overexpression had been induced in adulthood compared with G6OFF/ON mice (*p* = 0.0002, Figures 5C,D). We also found differences in calretinin levels in BG6ON animals in which GSK-3β had been overexpressed from birth (*p* = 0.018, Figures 5C,D). Taken together, these data demonstrate the activation of adult hippocampal neurogenesis in the BG6OFF/ON model.

### GSK-3β Overexpression Is Accompanied by a Reduction in Hippocampus-Dependent Memory, as Measured by the Object Recognition Test

The earliest distinctive feature of AD is short-term memory loss. The overexpression of GSK-3β from birth leads to cognitive impairment (Hernández et al., 2002). Also, memory can be restored in 6-month-old animals by inducing the shutdown of the system *via* the administration of doxycycline (Engel et al., 2006b).

**Figure 6:**
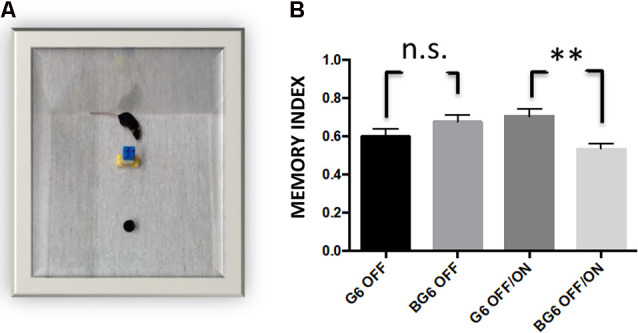
Effect of neuronal GSK-3β overexpression in adult mice on performance in the object recognition test (OR).** (A)** A representative image of the mouse recognizing the new object. **(B)** Two hours after familiarization, G6OFF, BG6OFF, and G6OFF/ON animals show the same ability to recognize the new object, while BG6 OFF/ON animals do not show memory retention. For quantifications, the data represented are the mean values with the SEM, and analyzed with two-tailed unpaired Student’s *t*-test; n.s. = not significant, **p < 0.001, *n* = 8–10 animals per group.

To study whether the histopathological alterations caused by GSK-3β overexpression in adulthood affected hippocampus-dependent memory, we subjected the mice to the OR test (Engel et al., 2006b). Significant differences in the performance of BG6OFF/ON animals compared with G6OFF/ON mice were detected (*p* = 0.0025, Figure 6). In this regard, the former spent almost the same time recognizing the new object as that devoted to exploring the object they had already been familiarized with. BG6 and G6 mice receiving doxycycline treatment for 12 months (system OFF) did not show any differences. These data would imply the loss of hippocampus-dependent non-spatial memory in the BG6OFF/ON model.

## Discussion

This article takes the analysis of the CamKIIα-tTA/Tet-O-GSK-3β mouse model (BG6) one step further (Lucas et al., 2001). In this regard, here we made use of the conditional nature of the system to repress the expression of the kinase during the first 6 months of life and have subsequently turned the system on by removing the doxycycline antibiotic from drinking water. This experimental approach allowed us to clarify whether previously observed effects in transgenic GSK-3β-overexpressing mice are due, in part, to adaptations that occur during development. This notion is especially relevant in the context of the dentate gyrus since the development of this structure occurs only in the postnatal period (Bayer and Altman, 1974).

Concerning the transgenic expression pattern directed by the CamKIIα promoter in adulthood (BG6OFF/ON animals), our results demonstrate that the system is turned on at 6 months of age, although the expression pattern differed from that observed in BG6ON animals. Thus, the former show lower expression of GSK-3β in the cerebral cortex, probably because the CamKIIα promoter is less active in this region of the brain during adulthood, as described for rats (Burgin et al., 1990). However, we found that the β-gal reporter protein showed high intensity both in the hippocampus and striatum. Of note, these first experiments demonstrated, as previously described in 12-month-old animals BG6ON, atrophy of the dentate gyrus (Engel et al., 2006a, Figure 1E). An increase in the number of caspase-3+ cells occurred in both BG6ON and BG6OFF/ON animals, thereby indicating that an increase in GSK-3β expression induces cell death by apoptosis. However, it seems that increasing GSK-3β in adult mice for 6 months (BG6OFF/ON) does not provoke dentate gyrus atrophy, suggesting that adult dentate gyrus is less susceptible to neurodegeneration or that at least 12 months (as in BG6ON mice) are necessary to observe atrophy of the dentate gyrus.

Somatodendritic accumulation of hyperphosphorylated tau, an early event in the neurofibrillary degeneration present in AD (Braak and Braak, 1991) was observed in the BG6OFF/ON mice. This observation confirms the relationship between GSK-3β and tau protein but this time in adult animals in which the enzyme expression had not been increased during embryonic or postnatal development. We should emphasize that tau hyperphosphorylation takes place in a somatodendritic location and with endogenous tau and not under high human tau overexpression, as observed in numerous transgenic models of tauopathies. Therefore, our hyperphosphorylation model is more similar to the context found in AD and other tauopathies. The increase in tau phosphorylation in BG6OFF/ON mice probably leads to a decrease in the affinity of this protein for microtubules and its subsequent accumulation in the soma. Of note, phosphorylated tau levels are not increased in 12-month BG6ON animals, an observation that contrasts with what has been described in animals of 3–6 months of age (Lucas et al., 2001; Engel et al., 2006b). Tau hyperphosphorylation and death by apoptosis correlates with an increase in reactive microgliosis. It is not clear whether microglia contribute to the tau pathology by not clearing tau, or by releasing factors that exacerbate the pathology (Sarlus and Heneka, 2017). However, it is reasonable to think that this reactive gliosis generates an inflammatory environment that causes neuronal alterations, although this point has not been addressed in this study. Several mechanisms can explain the neuronal stress and death (revealed by reactive glia and caspase-3 staining) in the BG6OFF/ON mice. Given the effects of GSK-3β overexpression on tau phosphorylation and compartmentalization, a possible mechanism could be the disorganization of the neuronal microtubule cytoskeleton. In this case, we propose that a decrease in microtubule stabilization caused by tau would lead to a reduction in microtubule content in our murine model, similar to that found in the brains of individuals with AD (Terry, 1998). The presence of phosphorylated tau does not predetermine the presence of aggregated tau. Thus, in this animal model, mouse tau will not aggregate or develop NFTs (Hernández et al., 2002) and BG6 mice recapitulate only some aspects of early AD. It is necessary to overexpress human tau protein with mutations present in patients with FTDP-17 to find tau protein in the form of filamentous structures (Engel et al., 2006c).

Overexpression of GSK-3β in transgenic mice induces learning deficits, as occurs in AD. In the present study, memory impairment in the young BG6ON animals (aged 3–6 months) measured by the Morris water maze test (Hernández et al., 2002) and the OR test (Engel et al., 2006b) were also reproduced in the BG6OFF/ON model. This observation thus confirms that the previous data (Hernández et al., 2002; Engel et al., 2006b) were not due to adaptation during brain development but rather to the direct effects of GSK-3β on hippocampus-dependent memory.

In the BG6OFF/ON model, adult hippocampal neurogenesis appears to be activated by a compensatory mechanism in response to insults that take place in the dentate gyrus, as described here by an increase in the number of DCX+ and calretinin+ cells. Our results show that this process can be reactivated in 12-month-old animals in which adult hippocampal neurogenesis is greatly diminished. However, based on the data presented, we do not know whether the DCX+ and calretinin+ neuroblasts can integrate into the hippocampal circuit. The data on the cognitive deficit obtained with the OR test suggests that this is not the case. Further work will allow us to clarify whether the increase in the number of DCX+ and calretinin+ cells is due to the enhanced division of the neuronal progenitors present in the subgranular region, or simply to a slowing of maturation, as we have described in young animals (Fuster-Matanzo et al., 2013).

Recent years have witnessed significant progress towards the generation of transgenic AD models, particularly about plaque formation and the toxic cascade of the β-amyloid peptide (Myers and McGonigle, 2019). However, if the deregulation of GSK-3β is a key event in the pathogenesis of AD, BG6 mice emerge as an alternative and/or complementary model. The most effort made to date on transgenic models of AD has focused on mimicking the neuropathological features of the disease and this may require excessively artificial modifications to reproduce within the life of a mouse something that is formed over many years in the human. Alternatively, it may simply not be possible to mimic all aspects of the neuropathology of AD in mice because specific metabolisms present only in humans are required [such as differences in microglia between humans and mice (Galatro et al., 2017) or in the sequence of the tau protein (Hernández et al., 2020)]. GSK-3β is an enzyme that is found at the convergence of the pathways involved in tau hyperphosphorylation, β-amyloid-induced toxicity, and PS-1 mutations. Compared to the existing mouse models of AD, BG6 mice are unique in that they reproduce intraneuronal dysfunction, which may ultimately be responsible for some aspects of neurodegeneration. Here we show that increasing GSK-3β activity in adult animals reproduces the first signs of AD, at least about tau protein and cognitive deficit.

In summary, our results demonstrate that the overexpression of GSK-3β in neurons only in adult animals and not during development reproduces the histopathological marks of AD, as well as the cognitive deficit associated with this neurodegenerative disease.

## Data Availability Statement

The raw data supporting the conclusions of this article will be made available by the authors, without undue reservation.

## Ethics Statement

The animal study was reviewed and approved by Consejería de Medioambiente de la Comunidad de Madrid (PROEX-412/15).

## Author Contributions

All authors listed have made a substantial, direct and intellectual contribution to the work, and approved it for publication.

## Conflict of Interest

The authors declare that the research was conducted in the absence of any commercial or financial relationships that could be construed as a potential conflict of interest.

## Supplementary Material

FIGURE S1 Schematic diagram of the experimental design. **(A)** BG6 mice are generated by crossing mice expressing tTA under control of the CamKIIα promoter (tTA, line B) with mice that have incorporated the BitetO construct in their genome (line G6). The double transgenic progeny (BG6) expresses GSK-3β constitutively in the brain unless doxycycline is given orally thus preventing transactivation by tTA. **(B)** Schematic representation of GSK-3β transgene expression in untreated mice (H_2_O) and transgene shutdown by doxycycline administration.

FIGURE S2 **(A)** β-galactosidase (β-gal) expression with GAPDH as a loading control for the cortex of the animals tested. The histogram shows β-gal/GAPDH levels in the different genotypes and conditions used. Note the significant increase in the hippocampus in BG6ON mice (*p* = 0.016) and BG6OFF/ON mice (*p* = 0, 024) respect to their G6 controls (*n* = 3 animals per group). **(B)** Expression of β-gal in the cortex and striatum. Images obtained for immunohistochemistry against the β-gal marker in BG6ON and BG6OFF/ON animals. For quantifications, the data represented are the mean values with the SEM and analyzed with a two-tailed unpaired Student’s *t*-test. **p* < 0.05; scale bars = 200 μm.
