# Supplementary figures and images for: Overexpression of GSK-3β in Adult Tet-OFF GSK-3β Transgenic Mice, and Not During Embryonic or Postnatal Development, Induces Tau Phosphorylation, Neurodegeneration and Learning Deficits
Source: Front Mol Neurosci. 2020 Sep 10;13:561470. doi: 10.3389/fnmol.2020.561470 (PMC7511757; doi:10.3389/fnmol.2020.561470)

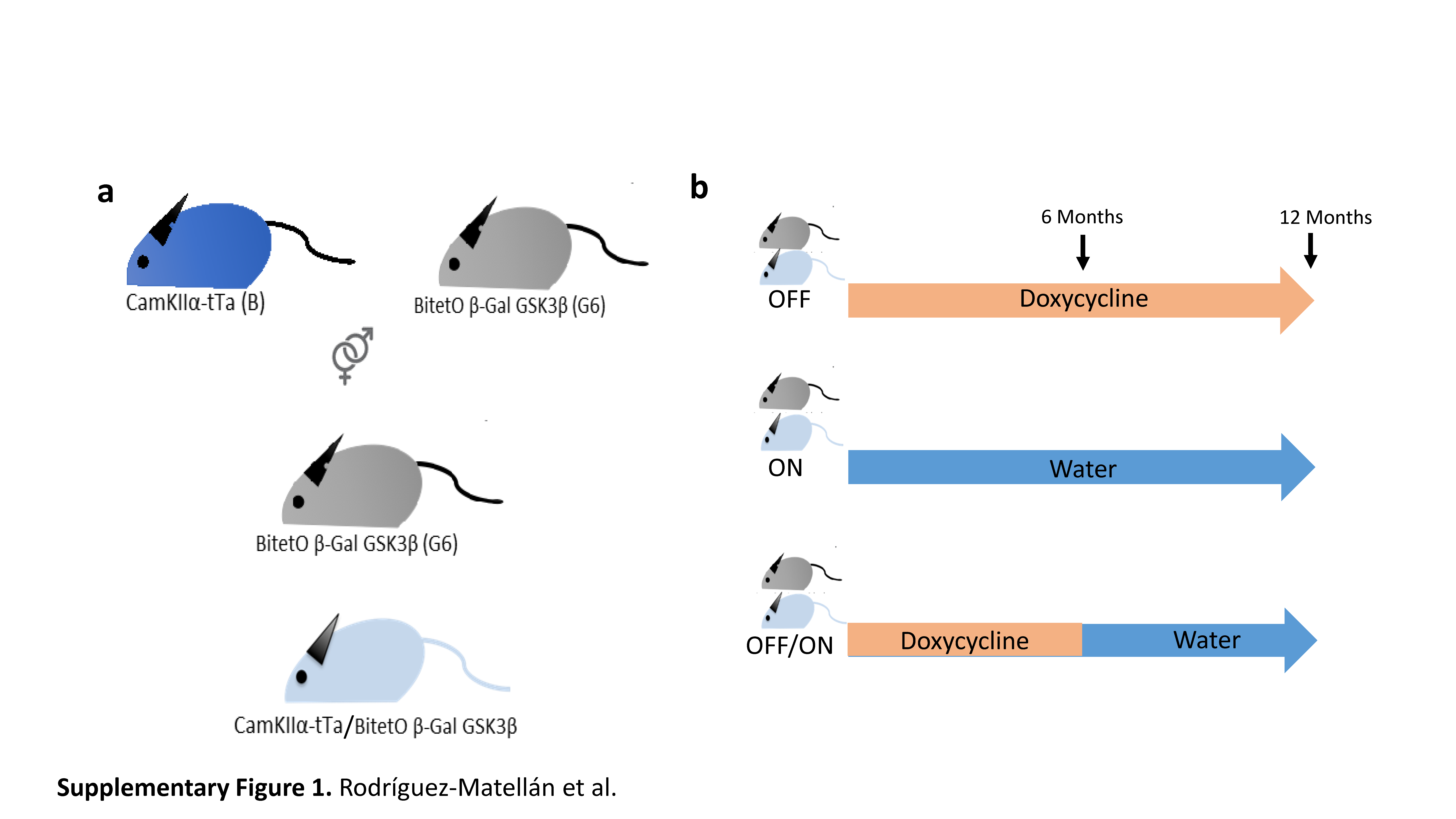

Supplement: FIGURE S1 — Schematic diagram of the experimental design. (A) BG6 mice are generated by crossing mice expressing tTA under control of the CamKIIα promoter (tTA, line B) with mice that have incorporated the BitetO construct in their genome (line G6). The double transgenic progeny (BG6) expresses GSK-3β constitutively in the brain unless doxycycline is given orally thus preventing transactivation by tTA. (B) Schematic representation of GSK-3β transgene expression in untreated mice (H2O) and transgene shutdown by doxycycline administration. [file Image_1.TIFF]

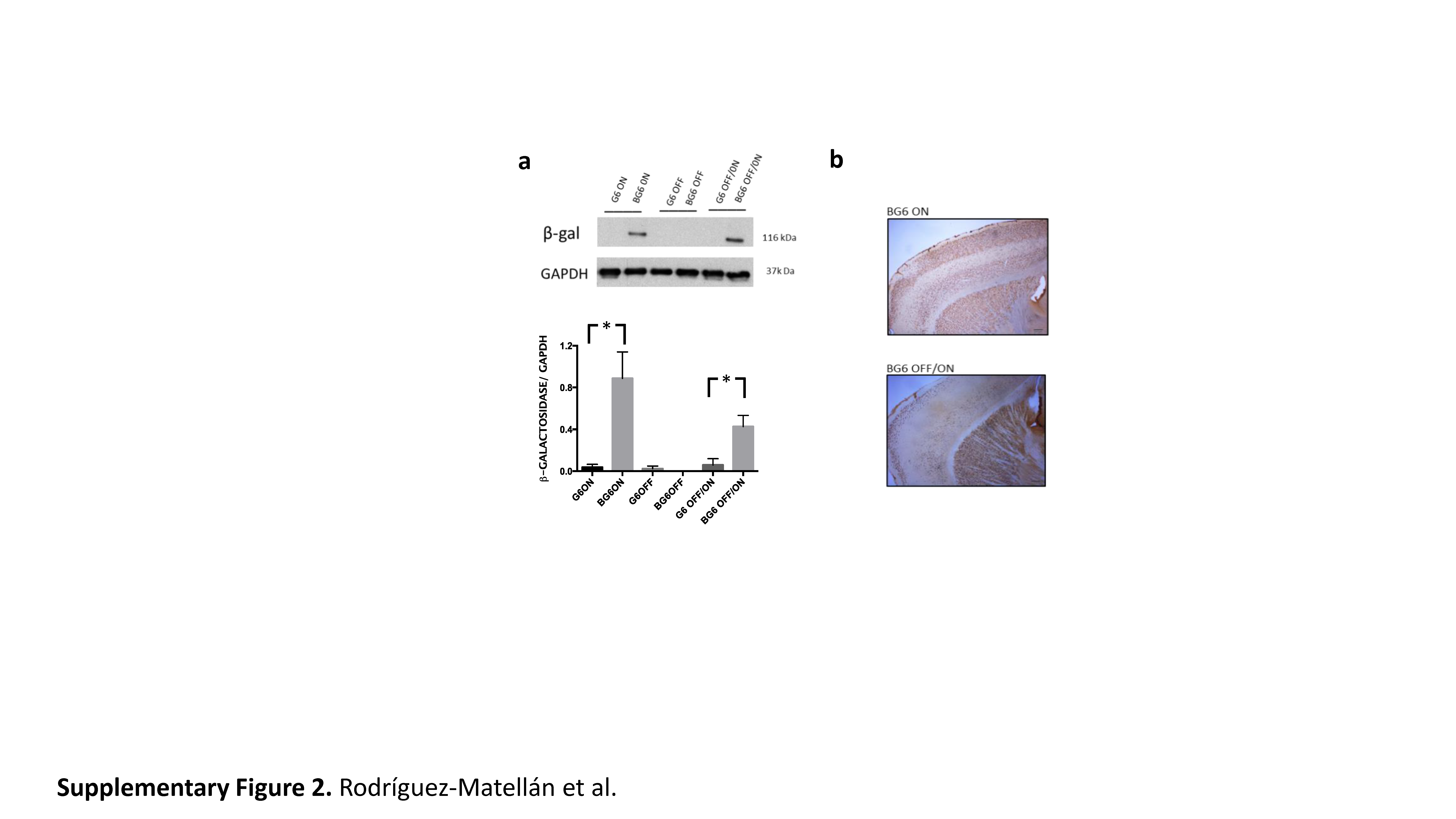

Supplement: FIGURE S2 — (A) β-galactosidase (β-gal) expression with GAPDH as a loading control for the cortex of the animals tested. The histogram shows β-gal/GAPDH levels in the different genotypes and conditions used. Note the significant increase in the hippocampus in BG6ON mice (p = 0.016) and BG6OFF/ON mice (p = 0, 024) respect to their G6 controls (n = 3 animals per group). (B) Expression of β-gal in the cortex and striatum. Images obtained for immunohistochemistry against the β-gal marker in BG6ON and BG6OFF/ON animals. For quantifications, the data represented are the mean values with the SEM and analyzed with a two-tailed unpaired Student’s t-test. *p < 0.05; scale bars = 200 μm. [file Image_2.TIFF]
